# Hypertension, BMI, and cardiovascular and cerebrovascular diseases

**DOI:** 10.1515/med-2021-0014

**Published:** 2021-01-21

**Authors:** Wenjing Qiao, Xinyi Zhang, Bo Kan, Ann M. Vuong, Shanshan Xue, Yuzheng Zhang, Binbin Li, Qianqian Zhao, Dingjie Guo, Xue Shen, Shuman Yang

**Affiliations:** Department of Epidemiology and Biostatistics, School of Public Health, Jilin University, 232-1163 Xinmin Street, Changchun, 130021, Jilin, China; Department of Noninfectious Chronic Diseases Control, Disease Prevention and Control Center, Fushun, Liaoning, China; Department of Clinical Laboratory, The Bethune Second Affiliated Hospital, Jilin University, Changchun, Jilin, China; Department of Epidemiology and Biostatistics, School of Public Health, University of Nevada, Las Vegas, United States of America

**Keywords:** cardiovascular and cerebrovascular diseases, body mass index, hypertension

## Abstract

Hypertension is associated with body mass index (BMI) and cardiovascular and cerebrovascular diseases (CCDs). Whether hypertension modifies the relationship between BMI and CCDs is still unclear. We examined the association between BMI and CCDs and tested whether effect measure modification was present by hypertension. We identified a population-based sample of 3,942 participants in Shuncheng, Fushun, Liaoning, China. Hypertension was defined as any past use of antihypertensive medication or having a measured systolic/diastolic blood pressure ≥130/80 mm Hg. BMI was calculated from measured body weight and body height. Data on diagnosed CCDs were self-reported and validated in the medical records. We used logistic regression models to estimate odds ratios (ORs) and 95% confidence intervals (CIs) for associations between BMI and CCDs. Higher BMI was associated with increased odds of having CCDs (OR = 1.19, 95% CI: 1.07–1.31). This association was significantly modified by hypertension (*P* for interaction <0.001), with positive associations observed among hypertensive individuals (OR = 1.28, 95% CI: 1.14–1.42). Age, sex, and diabetic status did not modify the relationship between BMI and CCDs (all *P* for interaction >0.10). Although higher BMI was associated with increased odds of CCDs, the relationship was mainly limited to hypertensive patients.

## 1 Introduction

Cardiovascular and cerebrovascular diseases (CCDs) are major public health problems worldwide [1,2,3,4], with 121.5 million individuals suffering from cardiovascular disease and ∼10.3 million new stroke cases during 2016 [5]. The incidence of CCDs is predicted to triple over the next few decades [6]. Stroke and ischemic heart disease were the top two causes for years of life lost globally in 2013 [7]. The total cost for hospitalization of acute myocardial infarction and stroke was 905.3 billion RMB (approximately US $137.4 billion) in 2016 [5]. In 2016, the number of deaths related to CCDs reached 17.9 million, accounting for 31% of all deaths worldwide [8].

It is well documented that higher body mass index (BMI) is associated with higher risk of CCDs [9]. Whether the relationship is modified by hypertensive status is unknown. For individuals with BMI ≥25 kg/m^2^, each 5 kg/m^2^ increase in BMI was associated with 40% higher risk of CCDs [9]. In addition, BMI is positively correlated with blood pressure [10]. For every 3 kg/m^2^ increase in BMI, the risk of hypertension increases by 50% in men and 57% in women [11]. Individuals with a BMI ≥ 24 kg/m^2^ have 3–4 times the risk of hypertension compared to individuals with a normal BMI in the general population [12]. Meanwhile, a prospective study in a Chinese population reported that for every 10 and 5 mm Hg increase in systolic blood pressure (SBP) and diastolic blood pressure (DBP), respectively, the risk of CCDs increased by 49 and 46% [13].

Hypertension may modify the association between BMI and CCDs, but there are few studies that have examined this [14,15]. Therefore, the present study sought to (1) examine the relationship between BMI and CCDs and (2) determine whether this relationship is modified by hypertensive status.

## 2 Materials and methods

### 2.1 Study population

Using a random sampling method, this cross-sectional study enrolled 4,553 population-based residents in Shuncheng, Fushun, Liaoning, China from 2013 to 2016. Shuncheng is a district located in northern Fushun, a northeastern city in China approximately 742 km away from Beijing. The area of Shuncheng is 348 km^2^, with a population of ∼0.4 million. This study randomly selected eight regions in Shuncheng and distributed 600 questionnaires in each region, totaling 4,800 questionnaires. Written informed consent was obtained from all participants before study enrollment. Among 4,800 returned questionnaires, 247 were excluded from the study because they were incomplete or failed to meet our quality control standards following an assessment. Although the response rate was 100%, we were only able to use 94.9% of the returned questionnaires. All data were fully anonymized before analyses. The study was conducted in accordance with the Declaration of Helsinki, and the protocol was approved by the Ethics Committee of Centers for Disease Control (CDC) of Fushun.

For the present study, we included all participants aged 18 years or older. We excluded individuals with missing or invalid data on body weight, height, status of CCDs, age, and sex.

### 2.2 Study measures

Our primary outcome was CCDs, which included cardiovascular diseases (e.g., coronary heart disease, angina, etc.) and cerebrovascular diseases (e.g., cerebral hemorrhage, cerebral thrombosis, etc.). CCDs were further categorized into cardiovascular diseases and cerebrovascular diseases. Information for CCDs was obtained via self-reported questionnaire inquiring whether they were ever diagnosed by specialists from government-funded hospitals. We also validated all reported diagnoses of CCDs based on their medical records.

The covariates for this study included age, body weight, body height, sex, hypertensive status, smoking, alcohol use, and diabetic status. Body weight, without shoes, was measured (to the nearest 0.1 kg) on an electronic scale. Body height, without shoes and in light clothing, was measured to the nearest 0.1 cm by a wall-mounted stadiometer. BMI was calculated using: body weight (kg) divided by body height squared (m^2^). The subjects were classified into the following categories according to their BMI: non-obese (<28 kg/m^2^) and obese (≥28 kg/m^2^); this classification was based on the 2002 recommendations of the CDC, China [16,17]. Each participant had his/her blood pressure measured after a period of rest of 5 min. SBP and DBP were measured with an automated sphygmomanometer thrice. Investigators asked each participant whether he/she had taken any antihypertensive medications. Hypertension was defined as any past use of antihypertensive medication or a having a measured SBP/DBP ≥130/80 mm Hg based on the guidelines released by the American College of Cardiology/American Heart Association (ACC/AHA) [18]. For our sensitivity analysis, we also defined hypertension as past antihypertensive medication use or SBP/DBP ≥140/90 mm Hg [19]. Fasting blood glucose was measured using a portable blood glucose meter. Diabetic status was defined as having a fasting blood glucose >7.1 mmol/L (http://www.diabetes.org/diabetes-basics/diagnosis/). A structured questionnaire was used to obtain sociodemographics as well as lifestyle and behavioral characteristics. Smoking was defined as current tobacco use. Individuals were classified as alcohol users if he/she reported a history of alcohol intake within the past 12 months.

### 2.3 Statistical analysis

In the descriptive analysis, continuous variables with a normal distribution are shown as means and standard deviations (SDs) and categorical variables were expressed as frequencies and percentages. The baseline characteristics of participants with and without CCDs were compared using independent *t*-test and chi-square test.

We used logistic regression models to estimate covariate-adjusted odds ratios (ORs) and 95% confidence intervals (CIs) for BMI (per SD increase) and CCDs. This model adjusted for age (continuous), sex (male/female), hypertensive status (yes/no), smoking (yes/no), alcohol use (yes/no), and diabetic status (yes/no). These covariates were selected *a priori* based on a literature review of the relationship between BMI and CCDs [5,8,20,21]. We further tested whether effect measure modification was present between BMI (per SD increase) and CCDs by the following variables: (1) age (≤50, >50), (2) sex (male/female), (3) diabetic status (yes/no), and (4) hypertension (yes/no). To do this, we built interaction terms between BMI and each variable in the logistic regression models. Interaction was considered to be present if the *P*-value was <0.05. All statistical analyses were performed using SPSS software (version: 24.0; SPSS, Chicago, IL, USA).

## 3 Results

**Figure 1:**
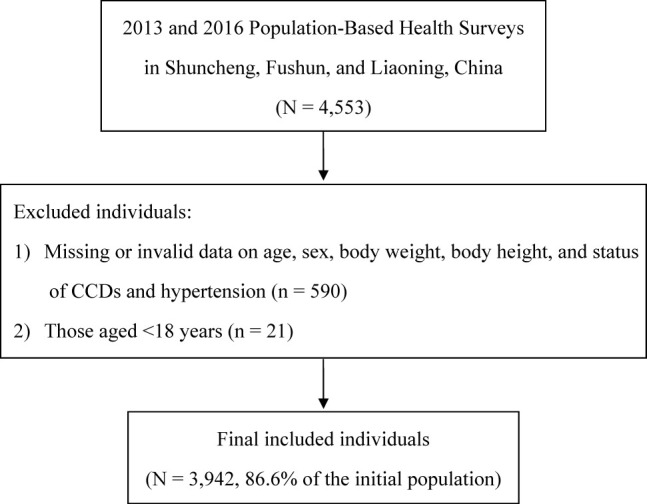
Flowchart for subject inclusion and exclusion.

Of the 4,553 individuals recruited, 3,942 individuals met our inclusion and exclusion criteria and were included in the present study (Figure 1). Average age of our study population was 48.9 years (SD = 15.9 years). There were 385 (9.8%) individuals with CCDs (Supplemental Table S1). Individuals with CCDs had significantly higher BMI, were older, more likely to be females, hypertensive (based on both hypertensive cut-points), and diabetic individuals (Table 1). The prevalence of hypertension based on a blood pressure of ≥130/80 and ≥140/90 mm Hg was 64.5 and 23.7%, respectively, in the overall study population. Smoking, alcohol use, and diabetes were observed in 24.0, 26.6, and 4.4% of the 3,942 study participants, respectively.

**Table 1:** Descriptive characteristics by status of cardiovascular and cerebrovascular diseases (CCDs)

Variable	With CCDs	Without CCDs	*P*-value
	*N* (%)	*N* (%)	
	(*n* = 385)	(*n* = 3,557)	
Age (years)	61.5 (11.5)	47.5 (15.7)	<0.001
BMI (kg/m^2^)	24.7 (4.1)	23.5 (3.5)	<0.001
Male (*n*, %)	154 (40.0)	1,801 (50.6)	<0.001
Hypertension (≥130/80 mm Hg) (*n*, %)	310 (80.5)	2,234 (62.8)	<0.001
Hypertension (≥140/90 mm Hg) (*n*, %)	197 (51.2)	737 (20.7)	<0.001
Smoking (*n*, %)	89 (23.1)	857 (24.1)	0.670
Alcohol use (*n*, %)	81 (21.0)	968 (24.6)	0.009
Diabetic status (*n*, %)	39 (10.1)	133 (3.7)	<0.001

Abbreviations: CCDs, cardiovascular and cerebrovascular diseases; BMI, body mass index. Values are presented as the mean (standard deviation) or frequency (percentage). Percentages have been rounded.

**Figure 2:**
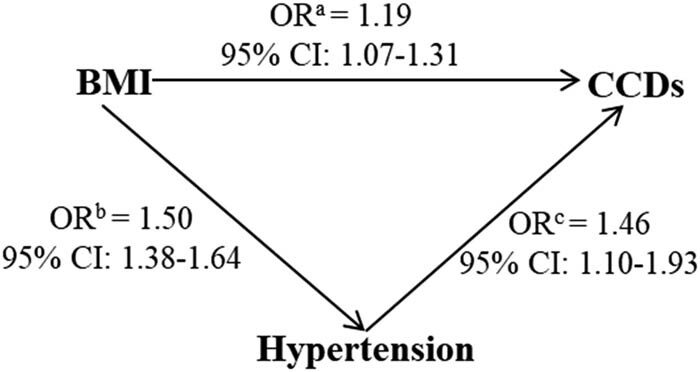
Associations between BMI per 1-SD increase, hypertension (≥130/80 mm Hg), and CCDs^a^. Abbreviations: CCDs, cardiovascular and cerebrovascular diseases; BMI, body mass index. The values are presented as the odds ratios (ORs) and 95% confidence intervals (95% CIs); ^a^adjusted for age, sex, hypertensive status, alcohol use, smoking, and diabetic status; ^b^adjusted for age, sex, alcohol use, smoking, and diabetic status; ^c^adjusted for BMI, age, sex, alcohol use, smoking, and diabetic status. The statistical analysis was performed with multivariate logistic regression analysis. The significance threshold was *P* <0.05.

After adjusting for covariates, a SD increase in BMI was associated with greater odds of having hypertension (≥130/80 mm Hg) (OR = 1.50, 95% CI: 1.38–1.64, Figure 2) and CCDs (OR = 1.19, 95% CI: 1.07–1.31). Hypertension (≥130/80 mm Hg) was associated with increased odds of CCDs (OR = 1.46, 95% CI: 1.10–1.93). Similar associations were noted when we further explored the relationship between BMI, hypertension (≥130/80 mm Hg), and cardiovascular diseases (Supplemental Figure S1) and cerebrovascular diseases (Supplemental Figure S2). Specifically, a SD increase in BMI was associated with increased odds of cardiovascular diseases (OR = 1.20, 95% CI: 1.07–1.34) but not with cerebrovascular diseases (OR = 1.03, 95% CI: 0.79–1.35) (Supplemental Table S2). Among individuals with BMI ≥28 kg/m^2^ and hypertension (≥130/80 mm Hg), the point estimate suggests a potential stronger relationship with CCDs than among individuals with a BMI <28 kg/m^2^ (Supplemental Table S3).

Significant effect measure modification by hypertension (≥130/80 mm Hg) was noted in the relationship between BMI and CCDs (*P* for interaction <0.001) (Table 2). The significant positive association between BMI and CCDs was only observed among individuals with hypertension (≥130/80 mm Hg) (OR = 1.28, 95% CI: 1.14–1.42) but not among non-hypertensive individuals (OR = 0.73, 95% CI: 0.55–0.99). Furthermore, findings indicate a significant inverse relationship between BMI and CCDs among non-hypertensive individuals. However, when hypertensive status was diagnosed as blood pressure ≥140/90 mm Hg, the interaction term between BMI and hypertension failed to reach statistical significance (*P* for interaction = 0.575). We did not observe interaction by age, sex, or diabetic status in the relationship between BMI and CCDs (all *P* for interaction >0.10).

**Table 2:** Adjusted odds ratios (ORs) and 95% confidence intervals (95% CIs) for associations between BMI (per 1-SD increase) and CCDs by age, sex, diabetic status, and hypertensive status

Variable	Subgroup	*n*	OR	95% CI	*P* for interaction
Age (years)	≤50	2,056	1.06	0.82–1.37	0.791
	>50	1,886	1.18	1.05–1.33	
Sex	Male	1,955	1.10	0.94–1.29	0.200
	Female	1,987	1.26	1.09–1.46	
Diabetic status	Yes	172	1.81	1.20–2.72	0.237
	No	3,770	1.10	0.99–1.23	
Hypertension (≥130/80 mm Hg)	Yes	2,544	1.28	1.14–1.42	<0.001
	No	1,398	0.73	0.55–0.99	
Hypertension (≥140/90 mm Hg)	Yes	934	1.17	1.01–1.34	0.575
	No	3,008	1.08	0.92–1.28	

Abbreviations: CCDs, cardiovascular and cerebrovascular diseases; BMI, body mass index. The values are presented as the odds ratios (ORs) and 95% confidence intervals (95% CIs); ORs were obtained after adjusting for age, sex, hypertensive status, alcohol use, smoking, and diabetic status.

## 4 Discussion

In this cross-sectional study, we found a significant positive relationship between BMI and CCDs. Interestingly, the positive relationship between BMI and CCDs was only found among hypertensive individuals (blood pressure ≥130/80 mm Hg). When hypertension was defined using ≥140/90 mm Hg, no interaction was noted for hypertensive status in the relationship between BMI and CCDs. These results suggested that early intervention and management of hypertensive individuals (blood pressure ≥130/80 mm Hg) with higher BMIs may be important to prevent CCD outcomes.

We found a significant association between BMI with cardiovascular disease but not with cerebrovascular disease. This is also in line with previous studies, in which BMI was more strongly associated with cardiovascular diseases than cerebrovascular diseases [22,23,24]. Huxley et al. and Barry et al. found that obesity was a risk factor for cardiovascular disease [22,24], whereas Sun et al. reported that the risk of cerebrovascular disease was not significantly different between overweight, obese, and normal-weight individuals [23].

In our study, we found that hypertension was more strongly associated with CCDs among obese individuals compared to non-obese people. These findings are consistent with previous studies [25,26,27]. As the baseline BMI level increases, individuals with hypertension have 2–3 times higher risk of CCDs than those with normal blood pressure [25]. It is likely that hypertension is an earlier predictor for CCDs among obese people.

We did not find significant interaction by age, sex, or diabetic in the association between BMI and CCDs. This is consistent with many previous studies, in which age, sex, and diabetes status were independent of BMI for assessing risk of CCDs [28,29,30].

We only found significant interaction by hypertension in the relationship between BMI and CCDs when hypertension was defined as having a blood pressure ≥130/80 mm Hg, but not ≥140/90 mm Hg. The underlying reasons for this finding are unclear. However, there is *in vitro* evidence suggesting that there are interrelated mechanisms between high blood pressure, BMI, and CCDs [14,15,31,32]. As this is the first epidemiological study suggesting these interrelationships, our findings warrant further confirmation.

The interrelationships between hypertension, BMI, and CCDs are likely to be attributed to numerous mechanisms. First, adipokines are highly deregulated under obesity and may control cardiovascular homeostasis [33,34]. Adipose tissue can release free fatty acids (FFA) in the proximity and around the coronary arteries, modulating vascular responsiveness to vasoactive agents [35] and turning into an adverse lipotoxic, pro-thrombotic, and pro-inflammatory factor (IFNγ) to overexpress chemotactic cytokines (i.e., MCP-1, IL-6) [33,36,37]. In addition, adipose tissue can discharge FFA into the bloodstream, disturbing vascular homeostasis and endothelial dysfunction, which leads to increased risk of CCDs [33,38]. Second, elevated blood pressure leads to systemic arteriole spasm by increasing the permeability of the vascular endothelium, prolonging the contact time of lipoproteins with the vascular wall, and reducing the endothelium-dependent vasodilation. The systemic arteriole spasm was suggested to increase risk of CCDs [39]. Third, blood pressure regulation is centered on endothelial function, which is regulated by the interaction of the renin–angiotensin–aldosterone system, adrenergic receptors, and metabolic reactions; these endothelial function-related mechanisms are also closely related to adipose tissue [40,41]. Furthermore, obesity-related FFA inhibits the sodium/potassium exchange pump and sodium-ATP pump, which increases smooth muscle tone, peripheral resistance, and blood pressure [42,43].

The present study’s findings have clinical implications at the population level. According to the latest ACC/AHA hypertension guidelines [18], we were able to screen more hypertensive patients compared to the previous standard (blood pressure ≥140/90 mm Hg). The advantage of using the new hypertension definition is that it will allow us to prevent adverse hypertension-related outcomes (i.e., CCDs) at an early stage.

There are several strengths for our study. First, we used a community-based population with random sampling methods. The possibility for generalizing our findings to the population is high. Second, this study had a high response and validity rate, further increasing the representativeness of the findings. Lastly, blood pressure for each participant was directly measured thrice at a stable and consistent condition. Antihypertension medications were also considered. All these factors ensured validity and reliability of hypertensive status.

Several limitations must be considered in the interpretation of our results. First, because of the cross-sectional study design, we cannot conclude a temporal relationship between hypertension and BMI with CCDs. Second, this study is conducted in a Shuncheng, Fushun, Liaoning, which is relatively a small area. Thus, the generalizability for our findings may be limited. Third, only some major CCD risk factors were considered in this study. Data for dietary intake, physical activity, and genetics were not included in this study, because they are not available. Potential residual confounding cannot be fully excluded.

In summary, there are interrelated relationships between BMI, CCDs, and hypertension. Furthermore, hypertension modifies the relationship between BMI and CCDs. Although BMI was independently associated with CCDs, this association was primarily limited to individuals with hypertension (≥130/80 mm Hg). These results suggested that prevention efforts for CCDs among obese individuals may need to focus on individuals with a blood pressure ≥130/80 mm Hg. Further studies are warranted to confirm our results.

## List of abbreviations

ACC/AHA: The American College of Cardiology/American Heart Association
BMI: body mass index
CCDs: cardiovascular and cerebrovascular diseases
CDC: Centers for Disease Control
CHD: coronary heart disease
95% CIs: 95% confidence intervals
DBP: diastolic blood pressure
FFA: free fatty acids
MI: myocardial infarction
ORs: odds ratios
RAAS: renin–angiotensin–aldosterone system
SBP: systolic blood pressure
SD: standard deviation
